# Mass COVID-19 testing of asymptomatic health-care workers in a tertiary hospital during an outbreak in another hospital in Singapore: an effective strategy?

**DOI:** 10.5365/wpsar.2022.13.4.951

**Published:** 2022-11-23

**Authors:** William T Wang, Hwang Ching Chan, Jyoti Somani, See Ming Lim

**Affiliations:** a Department of Medicine, National University Hospital, Singapore.

In response to Singapore’s first coronavirus disease (COVID-19) hospital outbreak from late April to early May 2021, we conducted a mass testing exercise on 3–7 May 2021. This cluster in a single hospital marked the arrival of the Delta (B.1.617.2) variant of severe acute respiratory syndrome coronavirus 2 (SARS-CoV-2) in Singapore, and was characterized by breakthrough infections in vaccinated health-care workers (HCWs) and some patients. (*1*) By late May, 47 cases of COVID-19 had been linked to this hospital cluster.

At the time of the hospital outbreak, close contacts of positive cases were identified and quarantined at government isolation facilities for 14 days. However, in light of concerns that contact tracing of positive cases alone may not prevent further transmission of SARS-CoV-2, especially by asymptomatic or very mildly symptomatic HCWs, all public hospitals were encouraged to conduct mass testing exercises as part of a strategy to minimise the risk of further spread to other hospitals.

This report describes the results of the national mass testing exercise in one public hospital, in an effort to assess the effectiveness of the mass testing of asymptomatic HCWs as a strategy to track and prevent viral spread through casual exposure to the Delta variant of SARS-CoV-2.

## Methods

Staff at our tertiary care hospital who had any contact with the source hospital at which the Delta variant outbreak occurred within 14 days of the mass testing exercise (3–7 May 2021) were invited to take a COVID-19 test and to complete a short online questionnaire. Participation was voluntary; staff who had already been identified as direct contacts through the Ministry of Health’s and the source hospital’s own extensive contact tracing programme were exempted. Staff were informed about the testing exercise by department heads and reporting officers, and invitations to participate were sent out via e-mail. Out of a total of approximately 7000 hospital staff, 427 indicated that they had had recent contact with the source hospital and attended for testing. Of the 427 tested, 165 presented to the staff clinic, while the remaining 262 attended the testing stations that were set up specifically for the purposes of the exercise. Nasopharyngeal swab samples were collected for polymerase chain reaction (PCR) testing for SARS-CoV-2.

The online questionnaire was designed to capture basic demographic data for each participant. Participants were also asked to provide information relating to their recent exposure history by selecting from a list of five possible exposure routes (multiple selections were allowed):

“I live in the same household as someone who works at the source hospital”

“I met with someone working on the source hospital campus for more than 30 minutes”

“I attended a meeting or training at the source hospital”

“I visited someone in the source hospital’s inpatient wards”

“I attended to patients or worked on the source hospital campus”

The data were anonymized by an independent third party and were retrospectively aggregated by the study team.

## Results

None of the 427 asymptomatic HCWs who participated in the testing exercise conducted at the tertiary hospital during 3–7 May 2021 tested positive for COVID-19. Of those tested, 163 (38.2%) reported living in the same household as a member of staff from the source hospital, 108 (25.3%) met with someone working on the source hospital campus for > 30 minutes, 59 (13.8%) attended a meeting or training at the source hospital, 18 (4.2%) visited someone in the source hospital’s inpatient wards and 16 (3.7%) attended to patients or worked on the source hospital campus during the time period of interest  (**Table 1**). Twenty-two (5.2%) reported more than one reason for exposure at the source hospital, while 41 (9.6%) went for testing without indication of any exposure.

**Table 1:** Self-reported sources of possible exposure to COVID-19 cases among staff at a tertiary care hospital who participated in a voluntary mass testing exercise (3–7 May 2021), as part of the response to an outbreak at another hospital, Singapore (*n* = 427)

Sources of possible exposure	“Yes” answers, *n*(%)
Lives in the same household as someone who works at the source hospital	163 (38.2)
Met with someone working on the source hospital campus for more than 30 minutes	108 (25.3)
Attended a meeting or training at the source hospital	59 (13.8)
Visited someone in the source hospital’s inpatient wards	18 (4.2)
Attended to patients or worked on the source hospital campus	16 (3.7)
More than one of the above	22 (5.2)
None of the above	41 (9.6)

## Discussion

Previous studies have suggested that mass testing of asymptomatic HCWs may help to reduce nosocomial transmission of COVID-19 by allowing early identification and isolation of positive cases, and contact tracing and quarantining of close contacts. (*2*) Additionally, the pre-symptomatic and early symptomatic periods have been identified as times of considerable transmission risk, with one study suggesting that more than 40% of cases may be infectious in the pre-symptomatic period. (*3*) The increased transmissibility of emerging variant strains of SARS-CoV-2 with shorter incubation periods adds further weight to the arguments in favour of employing HCW screening as a strategy to limit hospital transmission of COVID-19. (*4*)

In this relatively small, single-centre study, we did not detect a single case of COVID-19 in a group of 427 HCWs who submitted for PCR testing, despite the fact that 90.4% of participants reported possible exposure to someone from the source hospital. Our study sample excluded those HCWs who had a known exposure to a confirmed case at the source hospital and for this reason might be considered to be at greater risk of infection; however, all known contacts of positive cases at the source hospital also tested negative for COVID-19 on PCR swab tests.

Our testing exercise suggests that mass screening of asymptomatic HCWs is an ineffective strategy for preventing the spread of the Delta variant of SARS-CoV-2 in a hospital setting when there is a rapid and thorough contact tracing programme already in place. Any potential benefits would need to be weighed against any potential harms; for instance, a high proportion of negative tests may inadvertently result in complacency among hospital staff, leading to reduced compliance with infection control measures. Furthermore, implementing a mass testing exercise at any scale can be costly and may further exacerbate strained staffing and laboratory resources. (*5*) In many countries, increased infection rates among HCWs are typically proportional to increased infection rates within the community, suggesting that tracking community incidence to focus efforts on targeted screening may be more effective than conducting mass testing exercises. (*6*)

Limitations of this study include the possibility of incomplete capture of demographic and exposure history information; we mitigated against this by requiring staff to register and complete the online questionnaire before attending for their PCR test. In relying on self-reports to determine exposure through casual contact, our study will inevitably be subject to a degree of recall bias, especially in terms of the duration and level of exposure. Additionally, we acknowledge that some staff may have avoided the mass testing exercise despite known possible exposure. This was addressed by disseminating reminders through various communication channels to all staff about their obligation to public health safety and responsibility to participate in the mass testing if they had had any contact with the source hospital. Moreover, given the status of the pandemic at the time of our exercise (May 2021), motivation to be tested among staff was fairly high, prompted mainly by a desire to protect household members.

## Conclusion

Our mass testing exercise failed to uncover any instances of asymptomatic inter-hospital transmission. Within the limitations of this study, the results suggest that mass testing of asymptomatic HCWs may be an impractical infection control strategy to track and prevent COVID-19 transmission from one hospital to another. We suggest that an institutional testing strategy which mirrors local community incidence rates – whereby hospitals increase the frequency of asymptomatic testing when there is a surge in community cases but relax testing during periods of lower case rates – would be a more effective option, especially when combined with continued strict adherence to infection control measures and internal contact tracing and a recommendation that staff stay home when they feel unwell and report any cold/flu-like symptoms or close household exposures.
